# Reflections on Primary Health Care and Family Medicine in Botswana

**DOI:** 10.4102/phcfm.v6i1.648

**Published:** 2014-08-14

**Authors:** Vincent Setlhare

**Affiliations:** 1 Family Medicine, University of Botswana, Botswana

Primary Care should be the ‘central function and main focus of a country’s health system‘.^1^ The Ouagadougu Declaration similarly states that the vehicle for achieving the Millennium Development Goals and addressing the health of African nations is Primary Health Care (PHC).^2^

Dr. Margaret Chan, the Director General of the World Health Organization, addressing the 2013 World Congress of the World Organization of National Colleges, Academies and Academic Associations of General Practitioners and/or Family Physicians (WONCA), said that family doctors have always been the backbone of health care, and the bedrock of comprehensive, compassionate, people centred health care.^3^ Thus PHC and Family Medicine (FM) overlap and share the same principles^4^.

Botswana has a relatively small population of 2 038 228, which is unevenly distributed, with only 21% in urban settings.^5^ Nevertheless most of the people live within a 70 km radius of the capital, Gaborone, and more than 80% of the population live in a 100 km wide corridor on the south-east side of the country, bordering South Africa and Zimbabwe.^6^ Infectious diseases are the most common causes of morbidity and mortality in Botswana though non-communicable diseases are increasing.^6^ Infant mortality rates (IMR) and under five mortality rates are high, with death in the first week contributing 40% of IMR.^6^

Primary health care is not explicitly stated as the central function and main focus of Botswana’s health care system. Instead, Botswana seems to have adopted an ‘Integrated Health Services Plan‘ to meet the health needs of the nation through widespread and quality delivery of the ‘Essential Health Service Package‘.^7^ A variety of bodies are responsible for healthcare policy and service delivery. It is not clear which one takes leadership in formulating a vision and policy for health services delivery in Botswana.

Primary health care is a proven health care delivery system that Botswana should unequivocally adopt.^1^ Like South Africa, Botswana needs a champion at the highest level to boldly ‘recommit to primary health care as the foundation of the country’s health system‘.^8^ Countries with proven PHC success should be our role models, instead of relying on rich donor countries with poorly developed PHC and unremarkable health indices. This may help to avoid wasting resources on unproven health care systems. Some southern African countries have had PHC successes that address problems similar to Botswana’s and we can learn from them.

Most patients in Botswana are seen in PHC facilities and the health budget should reflect this. PHC facilities throughout Botswana need adequate resources to optimise health care delivery. Most of these facilities are run by nurses who also consult and treat patients, although not all are nurse practitioners, who have been trained to consult and treat patients. The Department of Family Medicine, the School of Nursing, and Nursing Institutes can collaborate so that training for PHC is streamlined to cater for nurse practitioners, clinical assistants and family physicians. Training of large numbers of such health workers should commence immediately so as to effectively address the Millennium Development Goals and achieve quality PHC. This training should largely be in PHC settings and as close as possible to all resources that are necessary for quality training.

PHC facilities should be staffed with nurse practitioners, doctors who are trained as generalists, and relevant support staff to ensure quality care. Family physicians are a new cadre of health care worker (an expert generalist doctor with postgraduate training through a 4-year MMed degree) that should provide leadership in PHC facilities and family physicians’ career paths should be attractive and allow them progression to higher levels.

The FM post-graduate programme has been running for four years and we hope to graduate our first crop of family physicians at the end of 2014. After graduation they will be registered as specialists. The FM post-graduate programme has been challenged by failure to recruit adequate staff, and the spreading of the few staff in three different locations. Stellenbosch University’s assistance with learning materials, administration of examinations and teaching visits by its staff has been very helpful. However, the solution to the staffing problem is to hire our own graduates.

The Department of Family Medicine provides training to undergraduates in years 1, 2, 3 and 5. Students seem to enjoy their FM rotations and this will hopefully incline some of them to take up FM as a specialty. The first University of Botswana MBChB students will graduate in mid-2014.

Wise and efficient use of meagre resources is necessary. Having fantastic government buildings and vehicles in our towns, whilst many of the people live in shacks and struggle to access simple drugs in public health facilities, is misuse of the commonwealth. Population distribution and prudent use of funds should inform the types of facilities and other resources assigned to different communities.
